# Remote working and occupational stress: Effects on IT-enabled industry employees in Hyderabad Metro, India

**DOI:** 10.3389/fpsyg.2023.1069402

**Published:** 2023-03-29

**Authors:** K.D.V. Prasad, Rajesh Vaidya, Ridhi Rani

**Affiliations:** ^1^Symbiosis Institute of Business Management, Hyderabad, India; ^2^Constituent of Symbiosis International (Deemed University), Pune, India; ^3^Symbiosis Institute of Business Management, Nagpur, India

**Keywords:** remote working, job satisfaction, occupational stress, motivation, performance

## Abstract

In the present study, the researchers reported the results of an empirical study on remote working and occupational stress and their effects on employees’ job satisfaction, motivation, and performance. Remote working has three subscales: self-proficiency, technology, and teamwork. Intrinsic and extrinsic motivation subscales were included to assess employee motivation. A simple random sampling method was used to select the subjects who are employees of the IT-enabled industries in Hyderabad Metro. A total of 513 responses were obtained on the remote working subscales—the effect on the independent variables, namely, employee self-proficiency, technology, teamwork, and occupational stress, on the dependent variables, namely, job satisfaction, intrinsic motivation, extrinsic motivation, and performance. The measured Cronbach’s alpha was in the range of 0.64–0.77, other reliability statistics split-half (odd-even) correlation was in the range of 0.62–0.84, and theSpearman–Brown prophecy was in the range of 0.70–0.91, demonstrating the reliability and internal consistency of the research instrument. The general linear model results indicated that all the independent variables, namely, self-proficiency, teamwork, and Occupational stress, are statistically significant and influence the outcome variables. The general linear model results also indicated statistically significant age differences in the dependent variables; however, there were no statistically significant gender differences. Of the independent variables, self-proficiency influences job satisfaction, intrinsic motivation, and performance (*p* < 0.01); teamwork influences employee job satisfaction and extrinsic motivation (*p* < 0.01 and *p* < 0.05); and Occupational stress influences performance (*p* < 0.01), which are statistically significant and thus influence the outcome variables. The model predicted a statistically significant influence of age (*p* < 0.01) on all the dependent factors, namely, job satisfaction, intrinsic motivation, extrinsic motivation, and performance. The study revealed that remote working is one of the major factors causing anxiety and employee stress. The main reasons are the absence of interaction with peers, the absence of routine fun during breaks, and work–family conflicts. Another observation is that the absence of peer–employee interaction demotivates the employees as there is no competition among the employees during remote working. The authors recommend that organizations develop an integrated human resource policy and performance management system that addresses the issues of employee stress, remote working concerns, peer–employee interactions, and pandemic-type situations. As there are several factors such as occupational stress, job satisfaction, motivation, peer interactions, and remote working concerns, employee stress-coping strategies affect the performance of an employee. The multiple mediation analysis indicates no statistically significant influence of the mediator variables, i.e., occupational stress and job satisfaction, on performance through remote working.

## 1. Introduction

Remote work began in the 1970s with the adoption of working from home to save on commuting expenses due to continuously soaring gasoline prices (Choudhury, 2020). Several IT giants, such as Tata Consultancy Services, planned to continue remote work and expected about 75% of their staff to work remotely by 2025, and Gitland, Zapier, Infosys, and MobSquad started adopting remote working procedures. Remote working is working from a non-designated place or outside the office, a new phenomenon that was a fallout of the coronavirus disease 2019 (COVID-19) pandemic. Organizations across the globe directed employees to work remotely to mitigate pandemic infections and protect families. Although the pandemic has subsided in several countries, many companies still ask their employees to continue working remotely (Prasad et al., 2020a,b). The main reason is to achieve a win-win situation for both the organizations, which save resources like office space charges, transport charges, electricity bills, and other miscellaneous expenses and the employees who benefit by saving commute time and enjoying more family time. However, moonlighting, where employees take up an additional or second job, has emerged as a side effect of remote working. The motives are lack of interest from the current organization, demotivation, and earning additional income for the family (Jehan et al., 2021). Other effects of remote work are workplace isolation, communication deficits, absence of interaction with peers, teamwork, handholding of peers in routine work, and family conflicts (Prasad et al., 2020a,b; Mookerjee et al., 2022). Sometimes, employees experience more stress due to workplace isolation.

In the recent past, working from home has become a more common topic of discussion in the information technology (IT) and information technology-enabled services (ITES) industries. Several organizations believe that it saves employees’ commuting time and overhead resources and addresses the issues of physical office management. Organizations, wherever possible, have asked their employees to work remotely or in a hybrid mode with a few people working in the office on a rotational basis. The employees were connected through conference applications such as Zoom, BlueJeans, Teams, and other applications for routine meetings and discussions (Prasad and Mangipudi, 2020; Mookerjee et al., 2022). Some staff members suffered post-pandemic stress and experienced severe emotional turmoil, affecting their psychological well-being. Some organizations have modified their policies and procedures to include remote or flexible working as a mode of employment.

A Reimagine Work Employee Survey (Dec 2020–Jan 2021, *n* = 5,043 full-time employees who work in corporate or government settings) in the US by McKinsey & Company reported that employees want more certainty about post-pandemic working arrangements. The survey further reported anxiety among the employees about their post-pandemic work engagement, particularly in hybrid employment, where most of the employees felt that the policies and procedures were not clearly communicated. Organizations with clearer communications are reaping the benefits of the balanced psychological well-being of their employees with enhanced productivity. Poor communication results in anxiety and contributes to employee burnout. For the global economy, the loss of productivity due to poor mental health, including anxiety and burnout, might be as high as US$1 trillion per year (Alexander et al., 2021).

A cross-sectional study among 209 employees working in Italian public and private organizations investigated the impact of family–work conflict, social isolation, job autonomy, distracting environments, work engagement, and stress experienced when working from home. The results indicated a negative relationship between employee family–work conflict and social isolation. Self-leadership and autonomy are positively correlated with work-from-home productivity and employee engagement. Individual and work-related factors hindered and facilitated work from home during the pandemic outbreak (Galanti et al., 2021).

Another study, using a sample of 5,452 Finnish employees, reported factors associated with abrupt employee adjustment to remote work by examining work independence and job clarity, interpersonal trust and social isolation, perceived work disruption and work location, and technology for communication during remote work. The authors concluded that these factors, which are necessary for employees to adapt to remote work, need to be addressed by the management of an organization (van Zoonen et al., 2021). Empirical research using online questionnaires was conducted to capture job dynamics inside homes, lifestyles, and habit modifications among 567 respondents. The study measured the well-being of workers who have adapted to working remotely during the pandemic. The responses were subjected to the structural equation methodology using the partial least squares method with SmartPLS 3.3.3. The six model dimensions of human relations, emotions, well-being, family economics, routines and habits, and family life were statistically significant to reflect the index of perception. The results indicated the perceptional changes among workers due to the lockdown. The two dimensions of human relations and family economics were relevant for remote-working employees to adapt to the remote-working mode of employment (Alvarez-Torres and Schiuma, 2022).

### 1.1. Literature review

An employee working remotely operates outside a designated office-based work environment. The offer of remote work to an employee requires considerable consideration and trust between the employee and their peer, and the peer needs to approve the employee’s remote work request. The COVID-19 pandemic was one of the reasons that companies encouraged remote work to mitigate the spread of the infection and protect families. If planned appropriately, remote working can enhance employee productivity, creativity, and other benefits for the organization (Greenbaum, 2019). Employee workplace isolation negatively influences remote employees due to absence of peer-to-peer and face-to-face meetings that provide guidance and inspiration to complete complex tasks (Hickman, 2019).

Flores (2019) explored the challenges of working remotely, such as the communication mediums of the organization, the skills required for a remote employee, and the benefits of remote working for both employees and employers. This study reported that email is a major communication medium and that flexible working hours benefit remote workers. Work–home conflict, loneliness, delay in decision-making, social and family support, job autonomy, work overload, and self-proficiency are the major factors affecting remote work employees (Wang et al., 2021).

Social support is positively associated with mitigating the challenges of remote employees, work overload, and monitoring of work–home conflicts. Remote working raises some ethical issues as work–life balance impacts the quality of life, motivation, role conflict management, and the achievement of organizational goals. Although remote working can enhance employees’ work–life balance, there is still some ambiguity as to when remote working is flexible and potentially increases productivity and maintains gender equity (Sullivan, 2012). The impact of remote working on work–life balance has negative effects due to unscheduled working hours. However, some employees have reported enhanced job satisfaction and work–life balance while working remotely (Bellmann and Hübler, 2020). Meiryani et al. (2022) reported that the impact of remote working on employees is statistically significant, but transformational leadership has no effect on employee performance. Social unions, peer–employee relations, trust, and communication influence employee engagement and performance (Adhitama and Riyanto, 2020). The personal habits of employees, unscheduled work schedules, and ergonomic issues also influence the work–life balance of employees (Muralidhar et al., 2020).

Orsini and Rodrigues (2020) argued the role of psychological needs in motivating educators and the role of team leaders and their leadership styles as potential motivators, offering a framework that can help leaders contribute to optimizing the remote working environment and increasing motivation. Dryselius and Pettersson (2021) studied the teleworking effects of motivation, such as the lack of social interaction, work–life balance challenges, and ineffective digital meetings, which are some of the factors that affect the motivation of remote workers. Rietveld et al. (2021) examined the relationship between perceived competence, employee autonomy, intrinsic motivation, and employee productivity in the spring of 2020. The study reported enhanced autonomy and competence during remote work, with decreased intrinsic motivation and productivity. The structural equation model results of this study indicated that a decrease in productivity was explained by a decrease in motivation. This study also confirmed that switching to remote working overnight impacted employee morale and decreased motivation.

Tapani et al. (2022) investigated higher education staff’s experiences on relevant issues related to remote working caused by pandemic. This research was carried out both at the earliest stage of the pandemic (April 2020) and during the pandemic (November–December 2021). The remote working experiences were analyzed through the lens of Deci and Ryan’s self-determination theory, especially through the concept of relatedness. Within this framework, relatedness is described as one of the three basic psychological needs that affect health, well-being, and productivity. The two sets of data analyses revealed three categories of relatedness: (1) interaction among coworkers, (2) feelings of care, and (3) experiences of connectedness. The results showed that the experience of relatedness was severely challenged during the enforced work period. In the future, the need for relatedness needs to be addressed more deliberately under multilocational work conditions because remote working particularly affects experiences of relatedness. Positive experiences of relatedness can also be achieved in remote work conditions.

Mendoza et al. (2022) carried out a cross-sectional study to assess the readiness of faculty and staff to assess alternative work arrangements and study the implications of a hybrid workforce model. The study examined 219 faculty and 69 staff members of a higher education institute in Olongapo City, Philippines. The study was in terms of access to transportation, health conditions, information technology resources, connectivity, and their preferences in terms of alternative work arrangements. The majority of the employees were inclined to work from home, given the nature of their duties. Most of the employees preferred a hybrid work environment over full–time work from home. This research implies that the place of residence is father distance from college, then it is necessary for assigning work from home. The age and health conditions, available information technology resources, and preferences of the worker influence the remote working conditions of an employee.

Subha et al. (2021) carried out an investigation to study the impact of occupational stress on the mental health of remote working women in Information Technology in Bangalore Urban, India. The data were gathered from 400 responses using convenience sampling. The continued period of remote working affected the well-being of female employees, with physical distance, dread, and vulnerability being some of the factors affecting employee well-being. The exploratory factor analysis identified job insecurity, workload, a poor work environment, a lack of structure, and personal problems as the five main factors that cause occupational stress during remote working. The multiple regression analysis revealed that the aforementioned five occupational stress-causing factors affect the mental health of female employees and that there is a negative and statistically significant inverse relationship.

To study the impact of working remotely on factors like work impact due to unscheduled working hours, gender differences in working hours, family distractions while working from home, and the absence of colleagues, a structured questionnaire was designed and sent to eight diverse sectors of Indian industry. The questionnaires were sent to Management Consulting, Fast Moving Consumer Goods, BFSI (Banking, Financial Service, and Insurance), Information Technology, Manufacturing, Pharmaceuticals, Retail, and Telecom. The responses received from the eight industries were analyzed, which indicated that the average working hours are 7.3 h/day with a median of 8 h and the maximum recorded hours are 11 h/day. However, there were no statistically significant differences between pre-COVID-19 and COVID-19 working hours for remote employees. Furthermore, family distractions are negatively affecting employee output. The study further confirmed that colleagues’ presence is a major factor in preferring to work from the office.

### 1.2. Research gap

After a critical and thorough review of the literature, the authors could source several articles on remote working and performance and remote working and motivation from the US and other Western countries. However, the literature on remote working, Occupational stress, and its effects on intrinsic motivation, extrinsic motivation, employee performance, and job satisfaction is limited, and in India, no study has been conducted on the IT and ITES industries. Furthermore, the authors could not source a single study on mediating the effects of occupational stress on performance with remote working as an independent variable. Thus, to fill this gap, the researchers surveyed the IT and ITES industry employees in the metropolitan city of Hyderabad, India.

### 1.3. Research questions

- Do remote working employees in IT Enabled sector experience occupational stress and does the occupational stress affect motivation, job satisfaction, and performance?
- Are remote working components, such as technology, teamwork, and employee self-proficiency, statistically significant, and do they influence employees’ job satisfaction, motivation, and performance?
- Is occupational stress’ influence on performance statistically significant as a mediator variable?

#### 1.3.1. Purpose of the research

To study the effects of remote working on Information Technology-enabled services employees’ job satisfaction, intrinsic motivation, extrinsic motivation, and performance.

#### 1.3.2. Main objectives

- To examine if remote working employees experience any occupational stress and whether its influence on employee job satisfaction, motivation, and performance is statistically significant.
- To examine whether the influence of remote working factors like technology, teamwork, and employee proficiency on job satisfaction, motivation, and performance are statistically significant.

#### 1.3.3. Hypotheses

*H_01_*: The remote working component, employee self-proficiency, is not significant and does not influence job satisfaction, intrinsic and extrinsic motivation, and performance.

*H_11_*: The remote working component, employee self-proficiency, is statistically significant and influences job satisfaction, intrinsic and extrinsic motivation, and performance.

*H_02_*: The remote working component, technology, is not significant and does not influence job satisfaction, intrinsic and extrinsic motivation, and performance.

*H_12_*: The remote working component, technology, is statistically significant and influences job satisfaction, intrinsic and extrinsic motivation, and performance.

*H_03_*: The remote working component, teamwork, does not significantly influence job satisfaction, intrinsic and extrinsic motivation, and performance.

*H_13_*: The remote working component, teamwork, is statistically significant and influences job satisfaction, intrinsic and extrinsic motivation, and performance.

*H_04_*: Occupational stress is not significant and does not influence job satisfaction, intrinsic and extrinsic motivation, and performance.

*H_14_*: Occupational stress is statistically significant and influences job satisfaction, intrinsic and extrinsic motivation, and performance.

*H_05_* The mediating role of the mediator variables occupational stress and remote working and their indirect effect is statistically significant.

*H_05_* The mediating role of the mediator variables occupational stress and remote working and their indirect effect is not statistically significant.

## 2. Research methodology

**Figure 1:**
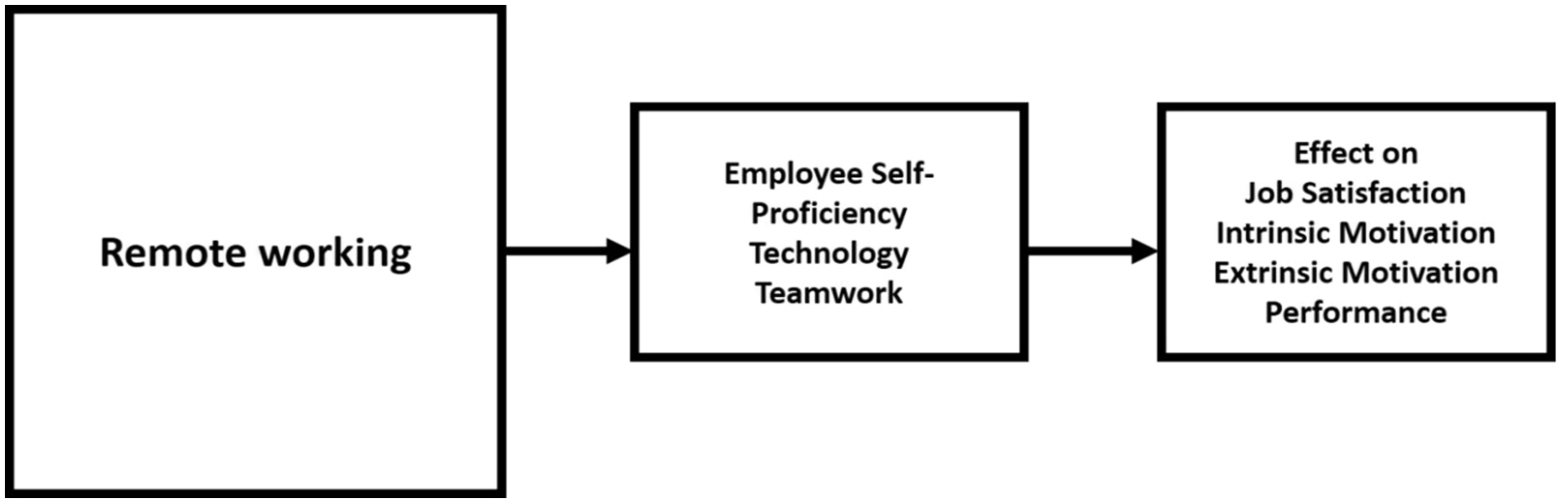
Theoretical framework-Remote work and job satisfaction, intrinsic and extrinsic motivation and performance.

The authors followed a theoretical framework based on the models proposed by Prasad et al. (2020a,b) and Muralidhar et al. (2020). The framework of this study is presented in Figure 1. The model assumed that, during remote working, employee self-proficiency, technology, and teamwork (predictor variables) will have significant effects on employee job satisfaction, intrinsic and extrinsic motivations, and employee performance (dependent variables). The data were gathered using a survey instrument—a structured questionnaire—to assess the effects of predictor variables on dependent variables. The questionnaire has 40 statements to measure the aforementioned seven factors, and the statements were systematically mixed to avoid any bias. The questionnaire was tested for reliability and validity and was found to be reliable and valid. This empirical study was carried out during May to August 2021, when the second wave of the COVID-19 pandemic was at its peak in India.

### 2.1. Determination of sample size

The total employee population in the IT and ITES sectors is not known, and we used the Cochran (1977) method to determine the sample size for this investigation. The minimum sample size required according to the Cochran, 1977 formula is 384. However, this study considered the responses of the 513 respondents who were employees in the IT and ITES sectors.

### 2.2. Research instrument and data collection

The simple random sampling method was used to select the subjects employed in the ITES industry in Hyderabad Metro. A list of 12,000 ITES industry employees was sourced, and each member was assigned a number. Using a random number generator software, a sample population of 600 subjects was selected. The link to the questionnaire was mailed to these 600 subjects. A total of 513 valid responses concerning the independent variables–employee self-proficiency, technology, teamwork and occupational stress; and on the dependent variables–job satisfaction, intrinsic motivation, extrinsic motivation, and performance were assessed.

Measuring the effects of remote working on IT employees: a five-point Likert-type rating scale, ranging from “Strongly agree = 5” to “Strongly disagree = 1,” was used to assess the remote working components of employee self-proficiency, teamwork, technology, and Occupational stress and their effects on job satisfaction, intrinsic motivation, extrinsic motivation, and employee performance based on the models proposed by Prasad et al. (2020a,b) and Muralidhar et al. (2020). The data were gathered by publishing a questionnaire on Google Forms and providing a link to all the respondents.

The demographic characteristics and age group details are presented in Tables 1, 2, respectively.

**Table 1:** Demographics of the sample.

	Frequency	Percent	Valid percent	Cumulative percent
Men	274	53.4	53.4	53.4
Women	239	46.6	46.6	100.0
Total	513	100.0	100.0	

**Table 2:** Age group of the study sample.

		Frequency	Percent
Valid	20–30 years	108	21.1
	31–40 years	176	34.3
	41–50 years	162	31.6
	51 years and above	67	13.1
	Total	513	100.0

The mean, standard deviation, skewness, and kurtosis of the study sample are presented in Table 3. The association between occupational stress and general characteristics is presented in Table 4.

**Table 3:** Mean, standard deviation, skewness, and kurtosis of the study sample.

	Mean	Standard deviation	Skewness	Kurtosis
Gender	0.466	0.499	0.137	−1.989
Age	2.367	0.957	0.11	−0.939
Self-proficiency	3.727	0.617	−0.875	0.682
Technology	3.682	0.661	−0.497	−0.468
Teamwork	3.690	0.598	−0.723	0.078
Job satisfaction	3.694	0.548	−1.113	1.303
Intrinsic motivation	3.876	0.577	−0.829	0.081
Extrinsic motivation	3.970	0.546	−0.848	0.568
Performance	3.710	0.431	−1.382	0.587
Occupational stress	3.286	0.691	0.184	−0.992

**Table 4:** Association between occupational stress and general characteristics.

	Frequency	Occupational stress	*p* value^*^
Gender			
Men	275	338	<0.00001
Women	239	175	
Are you married?			
Yes	138	338	<0.00001
No	375	175	
Do you have children?			
Yes	148	338	<0.00001
No	365	175	
Do you smoke?			
Yes	151	338	<0.00001
No	362	175	
Do you have diabetes?			
Yes	229	338	<0.00001
No	284	175	
Do you have hypertension?			
Yes	142	338	<0.00001
No	371	175	
Do you consume alcohol?			
Yes	101	338	0.000046
No	412	175	

Primary data; significant at 0.05 level.

*Values based on Chi-square.

Reliability statistics: Cronbach’s alpha, split-half (odd-even) correlation, and the Spearman–Brown prophecy were used to measure the internal consistency and reliability of the research instrument; the values are presented in Table 5. The values ranged from 0.64 to 0.77, 0.60–0.84, and 0.70–0.91, respectively, indicating that the research instrument maintained internal consistency and was reliable. The Spearman–Brown prediction formula (Spearman–Brown prophecy) is another type of reliability commonly used in survey research and also to test instrument reliability (Cronbach, 1951; Stanley, 1971; Allen and Yen, 1979; Wainer and Thissen, 2001). Split-half reliability using the Spearman–Brown formula was also used to test the reliability where the whole item was split into arbitrary halves, and the correlation between the split halves was converted into reliability by applying the Spearman–Brown formula (Kelley, 1924).

**Table 5:** Reliability statistics of the study sample.

Sl. No.	Study variable	Number of items	Split-half (odd-even) Correlation	Spearman-brown prophecy	Cronbach alpha
	Remote working (IV)				
1	Self-Proficiency	4	0.84	0.91	0.66
2	Technology	5	0.65	0.79	0.80
3	Teamwork	5	0.62	0.70	0.70
4	Occupational stress (IV)	5	0.77	0.88	0.84
5	Job satisfaction (DV)	5	0.68	0.77	0.64
6	Intrinsic motivation (DV)	4	0.80	0.89	0.69
7	Extrinsic motivation (DV)	4	0.77	0.87	0.67
8	Performance (DV)	8	0.67	0.80	0.69

## 3. Results

The hypotheses were tested using the general linear model -multivariate analysis using the Statistical Package for Social Sciences (SPSS version 28). The data were analyzed using a general linear model (GLM) multivariate analysis subjecting the independent variables, i.e., employee self-proficiency, technology, teamwork, and Occupational stress, against four dependent variables, i.e., job satisfaction, intrinsic motivation, extrinsic motivation, and employee performance. The results are presented below.

### 3.1. Test of equality of covariance matrices

This test reports that the assumption of the equality of the covariance matrices is satisfied. The sample size was large and the alpha value for this test was set at 0.001. The reported values in Table 6 are greater than (>) the set value. Therefore, that the assumption that equality of the covariance matrices of the outcome variable are equal across groups, is satisfied.

**Table 6:** Box’s test for equality of covariance matrices.

Box’s M	268.987
F	3.721
df1	70
df2	125531.296
Sig.	0.092

^a^Design: Intercept + Self-Proficiency + Technology + Teamwork + Occupational stress + Gender + Age Group + Gender * Age Group.

### 3.2. Levene’s test equality of error variances

The results in Table 7’s significance columns indicated that all the values are nonsignificant, the homogeneity of variance across the groups is equal, and the assumption of the equality of variance is not violated.

**Table 7:** Levene’s test for equality of error variances^a^.

	*F*	df1	df2	Sig.
Job satisfaction	7.252	7	505	0.123
Intrinsic motivation	1.724	7	505	0.101
Extrinsic motivation	1.682	7	505	0.111
Performance	2.401	7	505	0.095

^a^Design: Intercept + Self-Proficiency + Technology + Teamwork + Occupational stress + Gender + Age Group + Gender * Age Group.

The results of the multivariate tests are presented in Table 8. As the study met both the assumptions of Box’s test, the covariance matrices were equal, and Levene’s test of error variance indicated that the error variance was equal among the dependent variables. The authors also report Wilk’s Lamda results. The GLM results in Table 8 indicate the statistically significant results for the remote working components of employee self-proficiency (*p* < 0.01), teamwork (*p* < 0.01), and Occupational stress (*p* < 0.01), that affect employee job satisfaction, intrinsic and extrinsic motivation, and employee performance. The Wilk’s Lambda results for self-proficiency, teamwork, Occupational stress, and age were Wilk’s λ = 0.458, (F4, 498) = 5.497, *p* < 0.001, and η^2^ = 0.042; Wilk’s λ = 0.961, (F4, 498) = 5.073, *p* < 0.001, and η^2^ = 0.039; Wilk’s λ = 0.906, (F4, 498) = 12.882, *p* < 0.001, and η^2^ = 0.094; and Wilk’s λ = 0.458, (F12, 498) = 37.660, *p* < 0.001, and η^2^ = 0.229, respectively. There were no statistically significant gender differences affecting the dependent variables.

**Table 8:** General linear model: Multivariate tests^a^.

Effect		Value	F	Hypothesis df	Error df	Sig.	Partial Eta Squared
Intercept	Pillai’s trace	0.628	210.072^b^	4.000	498.000	<0.001	0.628
	Wilks’ lambda	0.372	210.072^b^	4.000	498.000	<0.001	0.628
	Hotelling’s trace	1.687	210.072^b^	4.000	498.000	<0.001	0.628
	Roy’s largest root	1.687	210.072^b^	4.000	498.000	<0.001	0.628
Self-Proficiency	Pillai’s trace	0.042	5.497^b^	4.000	498.000	<0.001	0.042
	Wilks’ lambda	0.958	5.497^b^	4.000	498.000	<0.001	0.042
	Hotelling’s trace	0.044	5.497^b^	4.000	498.000	<0.001	0.042
	Roy’s largest root	0.044	5.497^b^	4.000	498.000	<0.001	0.042
Technology	Pillai’s trace	0.006	.745^b^	4.000	498.000	0.561	0.006
	Wilks’ lambda	0.994	.745^b^	4.000	498.000	0.561	0.006
	Hotelling’s trace	0.006	.745^b^	4.000	498.000	0.561	0.006
	Roy’s largest root	0.006	.745^b^	4.000	498.000	0.561	0.006
Teamwork	Pillai’s trace	0.039	5.073^b^	4.000	498.000	<0.001	0.039
	Wilks’ lambda	0.961	5.073^b^	4.000	498.000	<0.001	0.039
	Hotelling’s trace	0.041	5.073^b^	4.000	498.000	<0.001	0.039
	Roy’s largest root	0.041	5.073^b^	4.000	498.000	<0.001	0.039
Occupational stress	Pillai’s trace	0.094	12.882^b^	4.000	498.000	<0.001	0.094
	Wilks’ lambda	0.906	12.882^b^	4.000	498.000	<0.001	0.094
	Hotelling’s trace	0.103	12.882^b^	4.000	498.000	<0.001	0.094
	Roy’s largest root	0.103	12.882^b^	4.000	498.000	<0.001	0.094
Gender	Pillai’s trace	0.016	2.084^b^	4.000	498.000	0.082	0.016
	Wilks’ lambda	0.984	2.084^b^	4.000	498.000	0.082	0.016
	Hotelling’s trace	0.017	2.084^b^	4.000	498.000	0.082	0.016
	Roy’s largest root	0.017	2.084^b^	4.000	498.000	0.082	0.016
Age Group	Pillai’s trace	0.564	28.928	12.000	1500.000	<0.001	0.188
	Wilks’ lambda	0.458	37.660	12.000	1317.876	<0.001	0.229
	Hotelling’s trace	1.134	46.916	12.000	1490.000	<0.001	0.274
	Roy’s largest root	1.090	136.215^c^	4.000	500.000	<0.001	0.521
Gender * Age Group	Pillai’s trace	0.012	0.520	12.000	1500.000	0.903	0.004
	Wilks’ lambda	0.988	0.519	12.000	1317.876	0.903	0.004
	Hotelling’s trace	0.013	0.519	12.000	1490.000	0.904	0.004
	Roy’s largest root	0.009	1.177^c^	4.000	500.000	0.320	0.009

^a^Design: Intercept + Self-Proficiency + Technology + Teamwork + Occupational stress + Gender + Age Group + Gender * Age Group.

^b^Exact statistic.

^c^The statistics is an upper bound on F that yields a lower bound on the significance level.

The GLM carried out a distinct analysis of variance for each dependent variable and independent factor. Table 9 shows the results of the test of between-subjects effects. It can be observed that the effect of employee self-proficiency was statistically significant and influenced the outcome variables as follows: Job satisfaction (*F*, 501) = 16.973, *p* < 0.001; and η^2^ = 0.033 and intrinsic motivation (*F*, 501) = 4.439, *p* < 0.05, and η^2^ = 0.009; teamwork was statistically significant and influenced the job satisfaction (*F*, 501) = 15.964, *p* < 0.001; and η^2^ = 0.031 and extrinsic motivation (*F*, 501) = 4.551, *p* < 0.05; and η^2^ = 0.009; and Occupational stress was statistically significant and influenced the performance (*F*, 501) = 46.950, *p* < 0.001; and η^2^ = 0.086; whereas age was statistically significant and influenced all the four dependent variables, namely, job satisfaction (*F*, 501) = 6.519, *p* < 0.001, η^2^ = 0.038; intrinsic motivation (*F*, 501) = 86.361, *p* < 0.001, and η^2^ = 0.341; extrinsic motivation (*F*, 501) = 140.869, *p* < 0.001, η^2^ = 0.485; and performance (*F*, 501) = 6.910, *p* < 0.001; and η^2^ = 0.040. The GLM has significantly predicted the outcome variables. Therefore, we partially accept the alternate hypotheses.

**Table 9:** General linear model multivariate analysis: Tests for between-subjects effects.

Source	Dependent variable	Type III Sum of Squares	df	Mean Square	F	Sig.	Partial Eta Squared
Corrected Model	Job satisfaction	34.771^a^	11	3.161	13.301	<0.001	0.226
	Intrinsic motivation	63.406^b^	11	5.764	26.993	<0.001	0.372
	Extrinsic motivation	74.122^c^	11	6.738	43.055	<0.001	0.486
	Performance	13.709^d^	11	1.246	7.687	<0.001	0.144
Intercept	Job satisfaction	28.915	1	28.915	121.672	<0.001	0.195
	Intrinsic motivation	72.995	1	72.995	341.830	<0.001	0.406
	Extrinsic motivation	81.687	1	81.687	521.944	<0.001	0.510
	Performance	39.574	1	39.574	244.074	<0.001	0.328
Self-Proficiency	Job satisfaction	4.033	1	4.033	16.973	<0.001	0.033
	Intrinsic motivation	0.948	1	0.948	4.439	0.036	0.009
	Extrinsic motivation	0.114	1	0.114	0.726	0.394	0.001
	Performance	0.601	1	0.601	3.705	0.055	0.007
Technology	Job satisfaction	0.100	1	0.100	0.421	0.517	0.001
	Intrinsic motivation	0.540	1	0.540	2.529	0.112	0.005
	Extrinsic motivation	0.076	1	0.076	0.483	0.487	0.001
	Performance	0.040	1	0.040	0.247	0.619	0.000
Teamwork	Job satisfaction	3.794	1	3.794	15.964	<0.001	0.031
	Intrinsic motivation	0.619	1	0.619	2.898	0.089	0.006
	Extrinsic motivation	0.712	1	0.712	4.551	0.033	0.009
	Performance	0.003	1	0.003	0.020	0.888	0.000
Occupational stress	Job satisfaction	0.044	1	0.044	0.187	0.666	0.000
	Intrinsic motivation	0.169	1	0.169	0.793	0.374	0.002
	Extrinsic motivation	0.009	1	0.009	0.058	0.810	0.000
	Performance	7.613	1	7.613	46.950	<0.001	0.086
Gender	Job satisfaction	0.128	1	0.128	0.538	0.464	0.001
	Intrinsic motivation	0.593	1	0.593	2.779	0.096	0.006
	Extrinsic motivation	0.136	1	0.136	0.871	0.351	0.002
	Performance	0.394	1	0.394	2.430	0.120	0.005
Age Group	Job satisfaction	4.648	3	1.549	6.519	<0.001	0.038
	Intrinsic motivation	55.325	3	18.442	86.361	<0.001	0.341
	Extrinsic motivation	66.141	3	22.047	140.869	<0.001	0.458
	Performance	3.361	3	1.120	6.910	<0.001	0.040
Gender * Age Group	Job satisfaction	1.057	3	0.352	1.482	0.219	0.009
	Intrinsic motivation	0.292	3	0.097	0.455	0.714	0.003
	Extrinsic motivation	0.015	3	0.005	0.032	0.992	0.000
	Performance	0.050	3	0.017	0.102	0.959	0.001
Error	Job satisfaction	119.060	501	0.238			
	Intrinsic motivation	106.984	501	0.214			
	Extrinsic motivation	78.409	501	0.157			
	Performance	81.233	501	0.162			
Total	Job satisfaction	7153.880	513				
	Intrinsic motivation	7876.313	513				
	Extrinsic motivation	8237.000	513				
	Performance	7154.219	513				
Corrected Total	Job satisfaction	153.831	512				
	Intrinsic motivation	170.390	512				
	Extrinsic motivation	152.532	512				
	Performance	94.942	512				

^a^*R* Squared = 0.226 (Adjusted *R* Squared = 0.209).

^b^*R* Squared = 0.372 (Adjusted *R* Squared = 0.358).

^c^*R* Squared = 0.486 (Adjusted *R* Squared = 0.475).

^d^*R* Squared = 0.144 (Adjusted *R* Squared = 0.126).

*H_01_*: The remote working component, employee self-proficiency, is statistically significant and influences job satisfaction, intrinsic and extrinsic motivation, and performance.

*H_02_*: The remote working component, technology, is not statistically significant and does not influence the job.

*H_13_*: The remote working component, teamwork, is statistically significant and influences job satisfaction, intrinsic and extrinsic motivation, and performance.

*H_14_*: Occupational stress is statistically significant and influences job satisfaction, intrinsic and extrinsic motivation, and job performance.

Statistically significant age group differences were observed, which influence all four outcome variables, namely, job satisfaction, intrinsic motivation, extrinsic motivation, and employee performance.

### 3.3. Multiple mediation analysis

The study examined the effect of more than one mediator on the performance of the dependent variable. The author followed the procedure of Preacher and Hayes (2008) which assessed the comparison of indirect effects, if any, in the multiple mediator models. This study examined the indirect effect of the mediator variables, i.e., job satisfaction and occupational stress on employee performance. Several studies have reported the mediating effect of job satisfaction on employee performance. A study by Gamal et al. (2022) with a sample of 86 employees at CV Purindah Lawang Malang reported that job satisfaction is a medium to improve the work environment’s effect on employee performance. A study on the mediating effects of job satisfaction and organizational commitment on self-reported performance by Vandenabeele (2009) provided more robust evidence of the relationship between motivation and performance in public service. In a study at the Department of Trade, Industry, and Cooperatives of Small and Medium Enterprises in Serang City with a population of 62 employees, data were analyzed for SEM analysis using SmartPLS. The results showed that job satisfaction has a positive and significant effect on employee performance (Alfarizi et al., 2022).

Based on the literature, the author included job satisfaction, and multiple mediation analysis was carried out using IBM SPSS Amos version 28. The authors used the estimands function of IBM SPSS Amos to estimate the mediating roles of job satisfaction and employee performance.

#### 3.3.1. Results of mediation analysis

The study assessed the mediating roles of job satisfaction and occupational stress on the relationship between remote working and employee performance. The results revealed that the insignificant indirect effect of occupational stress on job performance during remote working (*b* = 0.002, *t* = 0.50, and *p* = 0.375) does not support H5. Analyzing the mediating role of job satisfaction on the linkages between remote working and employee performance (*b* = 0.002, *t* = 0.40, and *p* = 0.267), job satisfaction was found to not support H6. Furthermore, the direct effect of remote working on employee performance in the presence of the mediators’ job satisfaction and occupational stress was found to be significant (*b* = 0.873, *t* = 12.671, and *p* = 0.000). Hence, job satisfaction and occupational stress are not mediating the relationship between remote working and employee performance. The mediation analysis summary is presented in Table 10.

**Table 10:** Summary of mediation analysis.

Relationship	Direct effect	Indirect effect	Confidence interval		*p* value	Conclusions
			Lower bound	Upper bound		
Remote working→job satisfaction→employee performance	0.873 (0.000)	0.002	−0.003	0.04	0.267	No mediation
Remote working→occupational stress→employee performance		0.002	−0.004	0.013	0.375	No mediation

Are any indirect paths significant?

**Figure 2:**
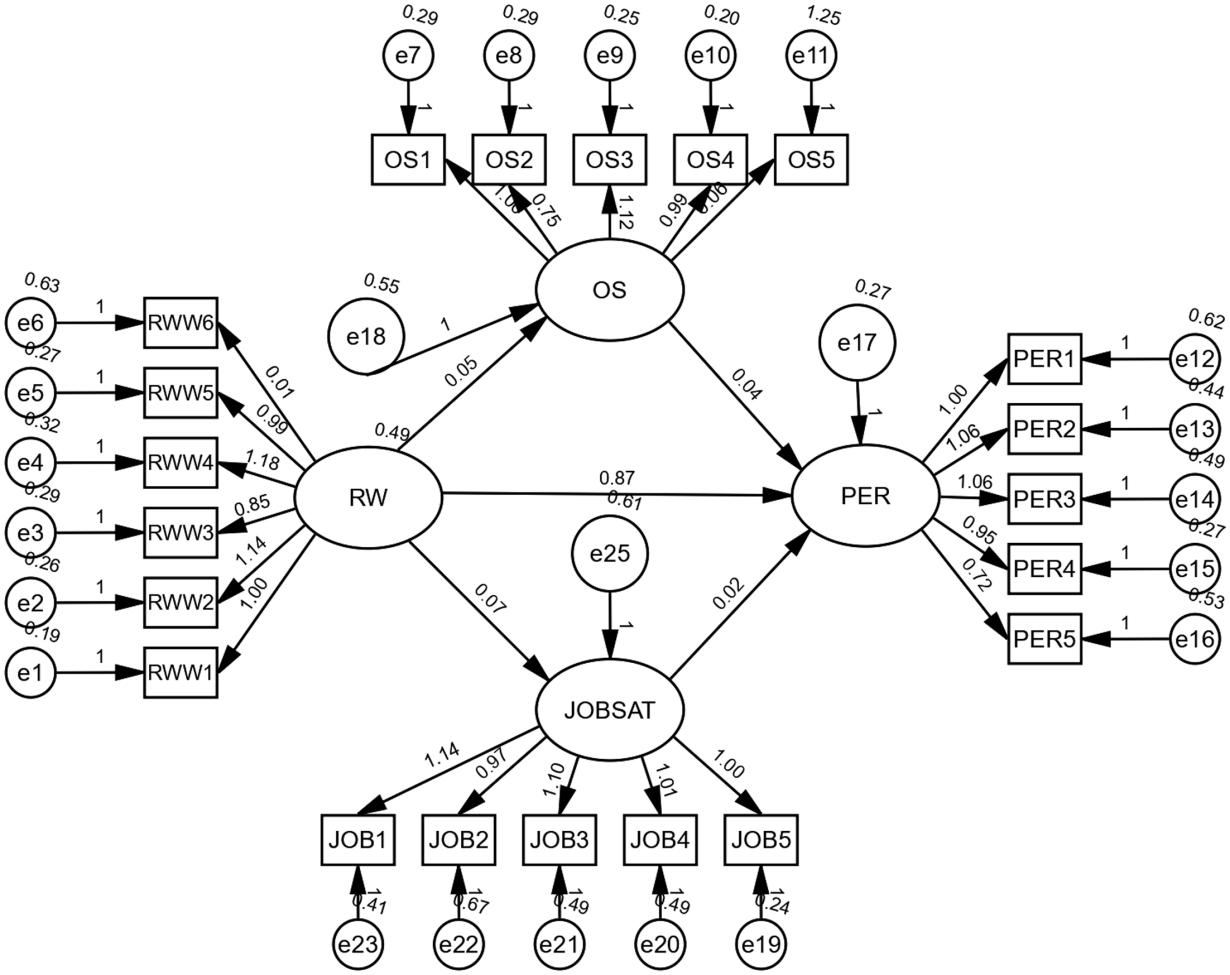
Mediation analysis. REMWOR, remote working; PERFO, performance; Stress, occupational stress; JOB, job satisfaction; RWW, remote working; OS, occupational stress; and PER, performance.

- a1b1 represents the indirect effect of remote working on employee performance through occupational stress (Figure 2).
- a2b2 represents the indirect effect of remote working on employee performance through job satisfaction.
- Based on these results, the authors can conclude that the influence of remote working on employee performance through job satisfaction and occupational stress is not significant.
- The direct effect of remote working on employee performance is significant (*b* = 0.873 and *p* = 0.000).

The results indicate no mediation of the mediating variables job satisfaction and occupational stress.

The total indirect effect of 0.001 is the sum of (a1*b1) and (a2*b2)

a1: remote working to occupational stress   0.048
a2: occupational stress to performance   0.042
a2: remote working to job satisfaction   0.074
b2: remote working to performance   0.022.

Therefore, a1*b1 = 0.048*0.042 = 0.002; a2*b2 = 0.074*0.022 = −0.002.

Therefore, we rejected the H5 null hypothesis and accepted the H5 alternate hypothesis.

## 4. Discussion

The research instrument, a 40-statement questionnaire deployed to measure the effect of predictor variables on dependent variables, was published on Google Forms. Approximately 550 responses were received. However, 37 responses were eliminated because they were incomplete, and 513 valid responses were subjected to GLM analysis. Several studies have been carried out on Occupational stress, remote working, and their effects on employee performance and motivation in the US and other Western countries. However, limited literature is available in this domain, particularly on remote working components such as technology, employee self-proficiency, teamwork, and stress and their effect on job satisfaction, intrinsic motivation, extrinsic motivation, and employee performance. The Cronbach’s alpha values, split-half (odd-even) correlations, and Spearman–Brown prophecy values indicate that the research instrument, i.e., the questionnaire, maintained internal consistency and reliability.

Chakraborty and Bhattacharya (2022) in their study on remote working in India collected data from eight different sectors and reported significant age group differences and no statistically significant gender group differences in remote working factors such as working hours and teamwork, which is in line with the outcomes of our studies in the Indian scenario.

Sultana et al. (2021) conducted a study on 400 homeworkers to explore motivation, commitment to job satisfaction, and employee performance from the remote working perspective. The results indicated that normative commitment and intrinsic motivation are the two most important variables that have a direct influence on both employees’ job satisfaction and their performance in the context of remote working. The results also revealed the partial mediating role of job satisfaction on employees’ performance through employee involvement. These results are similar to the findings of our study on factors such as job satisfaction, motivation, and employee performance.

Remote working has become the “new normal” for many business houses and institutes, engendering further challenges for employees who have started experiencing anxiety, technostress caused by digitalization and lack of social interaction, frustration, occupational burden, counterproductive work behavior, exhaustion, burnout, depersonalization, and increased turnover intentions with decreased motivation and job satisfaction. In this context, the authors carried out a study with 850 Romanian employees on the remote working concept and concluded that internal marketing significantly impacts job satisfaction. In turn, it impacts task performance and counterproductive work behavior. Job satisfaction is positively correlated with task performance (Nemteanu and Dabija, 2021). Our results are concurrent with this study on factors like job satisfaction, job performance, and employee motivation.

The authors used a modified five-point Likert-type scale to measure stress, components of remote work, and dependent variables such as job satisfaction, motivation, and employee performance. The results are in line with those of earlier studies carried out by Prasad et al. (2020a,b), which reported on occupational stress, remote working, and psychological well-being by Sonnenschein et al. (2022), which reported on employee motivation, job satisfaction, and working from home, and by Irawanto et al. (2021), which reported on working from home, job satisfaction, motivation, stress, and work-life balance.

### 4.1. Limitations of the study

The data were gathered from employees in the IT and ITES industries around Hyderabad Metro, India. The authors believe that the results can be generalized to some extent because the sample size is sufficiently large. The authors suggest similar studies across Indian cities so as to yield results that can help address stress-related issues among those who work remotely. More studies on variables such as time adjustment, maintaining good peer–employee relations, meditation, and yoga may be helpful to assess the mitigating and negative effects of stress on employee performance and work quality.

## 5. Conclusion

Remote working has become the “new normal” for many business houses and organizations, creating more challenges for employees, who are experiencing anxiety, stress due to technology, lack of social interaction and isolation, occupational load, counterproductive work behavior, fatigue, burnout, and enhanced turnover intentions. Although it has been more than 2 years since the occurrence of the COVID-19 pandemic, some organizations across the world still have a hybrid mode of work culture. To date, an employee cannot confidently perceive and express that working from home is convenient. Remotely carrying out the majority of the work is not an ideal way forward. There is an urgent need to define what sort and type of work can be done remotely or online. Further, the nature of the work can determine how easily and well it can be performed online. The organizations can analyze the current job assignments and come to a conclusion on remote working that suits the pandemic scenario. There is a need to change jobs to maximize output from remote working. The authors believe that several organizations are not prepared to work remotely as needed in certain environments. It is essential to have boundaries for employees’ distractions and make sure that they are minimal while working. Creating some designated workspaces near the employee residence or a separate workspace within the residence for working full-time at a stretch without distraction and disturbance is essential. The employees should also plan their daily activities and household work in advance and schedule them accordingly while remote working to maximize efficiency. If both family members (wife and husband) or more members are working remotely, the household activities can be divided based on their work schedules to not place the maximum burden on a single person.

Although several studies have been carried out on remote working and its effects on employees, the authors studied the effects of remote working and occupational stress on employee job satisfaction, intrinsic and extrinsic motivation, and performance in Hyderabad Metro, India, where more than 1 million employees work in IT and ITES. The study revealed the statistically significant influence of components of remote working such as employee self-proficiency and teamwork on job satisfaction, intrinsic and extrinsic motivation, and employee performance. Several studies reported increased occupational stress due to remote working, and our study confirms that Occupational stress is statistically significant and influences job satisfaction, intrinsic and extrinsic motivation, and job performance. Our results are in line with a study carried out by Prasad et al. (2020a,b) on the effects of Occupational stress and remote working on the well-being of employees during the COVID-19 pandemic, which involved surveying information technology employees around Hyderabad Metro. This study reported that the influences of remote working and occupational stress on job satisfaction and performance are statistically significant. Subha et al. (2021) reported that stress levels are high in women, in particular, women IT professionals working remotely. Moreover, there is a significant absence of downtime to reenergize as there is the fear of job insecurity.

## 6. Recommendations

Although remote working is not a choice of the employee but a necessity to mitigate the infection, one study implied that, wherever possible, remote working options should be explored by the employer, taking into account the nature of the jobs. Setting up designated workspaces near the residences of employees so small groups of employees from the same organization can work and interact reduces stress and enhances job satisfaction. Virtual offices, frequent peer interactions, and efficient post-COVID-19 pandemic back-to-workplace policies and procedures are the need of the hour. It is essential to set up small satellite offices closer to the localities of employees, as it benefits employees coming from suburban areas, rural hinterlands, and less-developed parts of the country. The deskless worker mode has also been proposed as a part of post-pandemic workplaces, whereby an individual will not attend the office every day and instead will perform their tasks, roles, and responsibilities from their own locations and spaces.

Employees’ performance depends not only on their work or job assignments but on several internal and external factors. Occupational stress, coping strategies, remote working, social support, psychological well-being, work–life balance, employee engagement, job satisfaction, and motivation factors will significantly influence employee performance (Prasad, 2021). Therefore, there is an urgent need to develop and implement an integrated new-age performance management framework that considers the said factors, as the traditional performance policies have lacunae and are traditionally assigned to the vision, mission, and goals of the organization without considering the said internal and external factors.

## Data availability statement

The original contributions presented in the study are included in the article/Supplementary material, further inquiries can be directed to the corresponding author/s.

## Ethics statement

Ethical review and approval was not required for the study on human participants in accordance with the local legislation and institutional requirements. Written informed consent from the (patients/participants OR patients/participants legal guardian/next of kin) was not required to participate in this study in accordance with the national legislation and the institutional requirements.

## Author contributions

KP: study design, data analysis, and writing. RV: overall manuscript editing, addressing reviewer’s concerns, and statistical procedures. RR: data collection and treatment associated with the data analysis. KP, RV, and RR conceptualized the contributions. All authors contributed to the article and approved the submitted version.

## Conflict of interest

The authors declare that the research was conducted in the absence of any commercial or financial relationships that could be construed as a potential conflict of interest.

## Publisher’s note

All claims expressed in this article are solely those of the authors and do not necessarily represent those of their affiliated organizations, or those of the publisher, the editors and the reviewers. Any product that may be evaluated in this article, or claim that may be made by its manufacturer, is not guaranteed or endorsed by the publisher.
